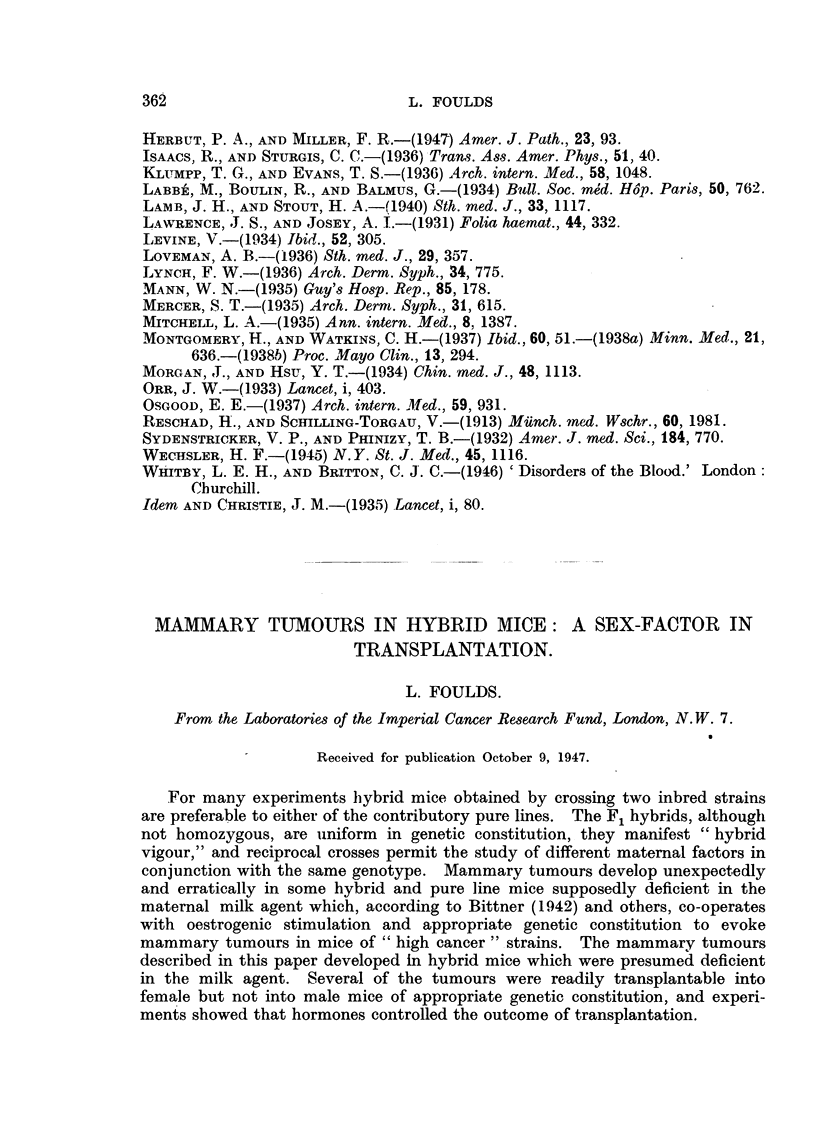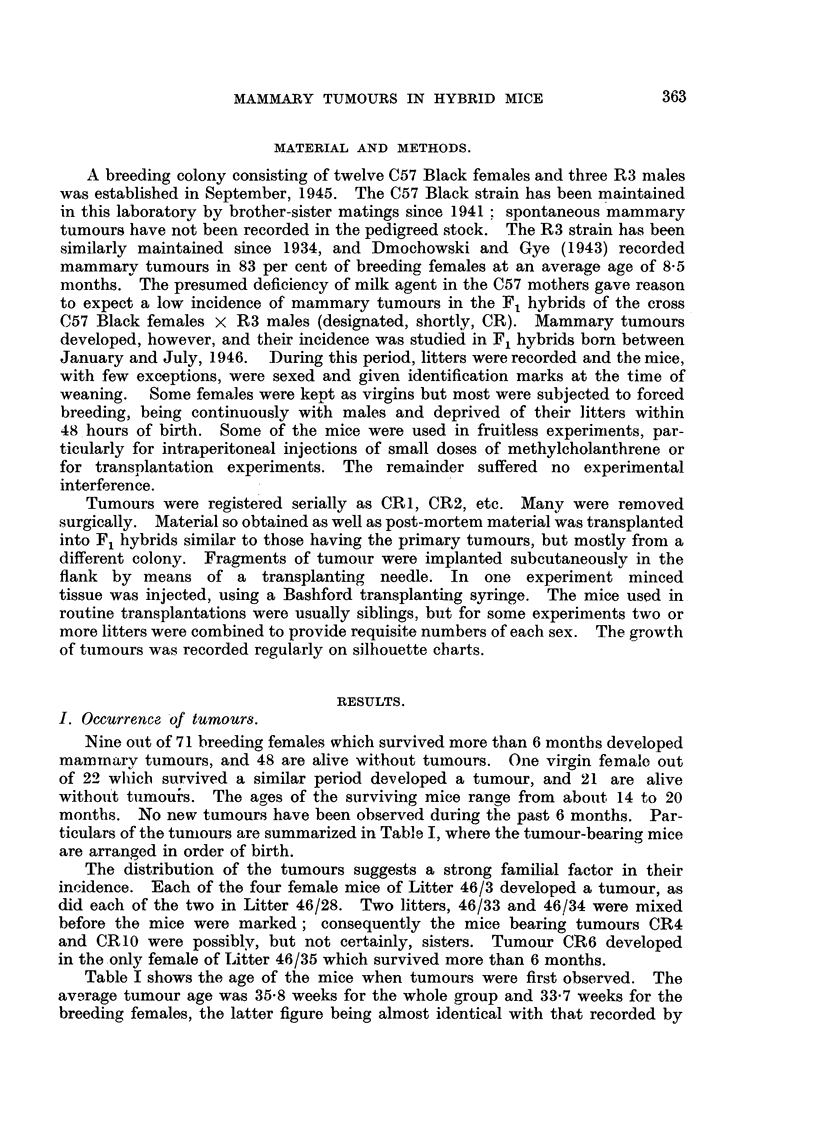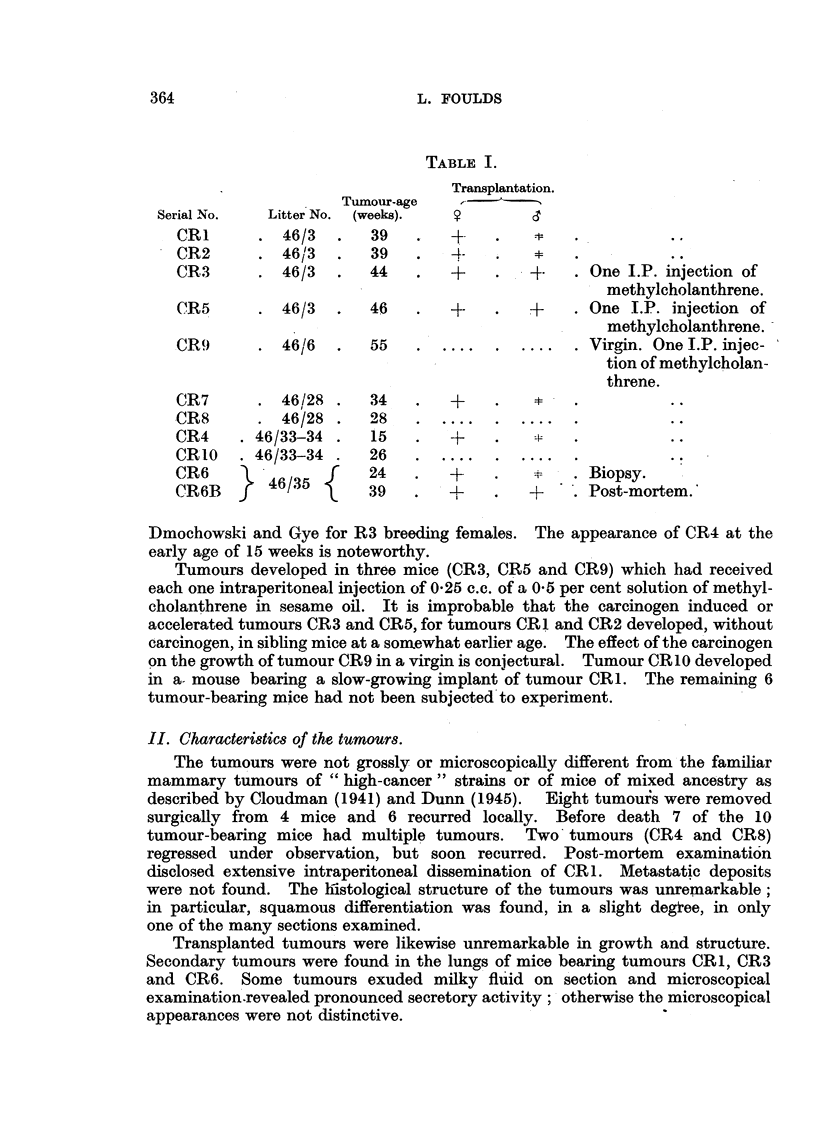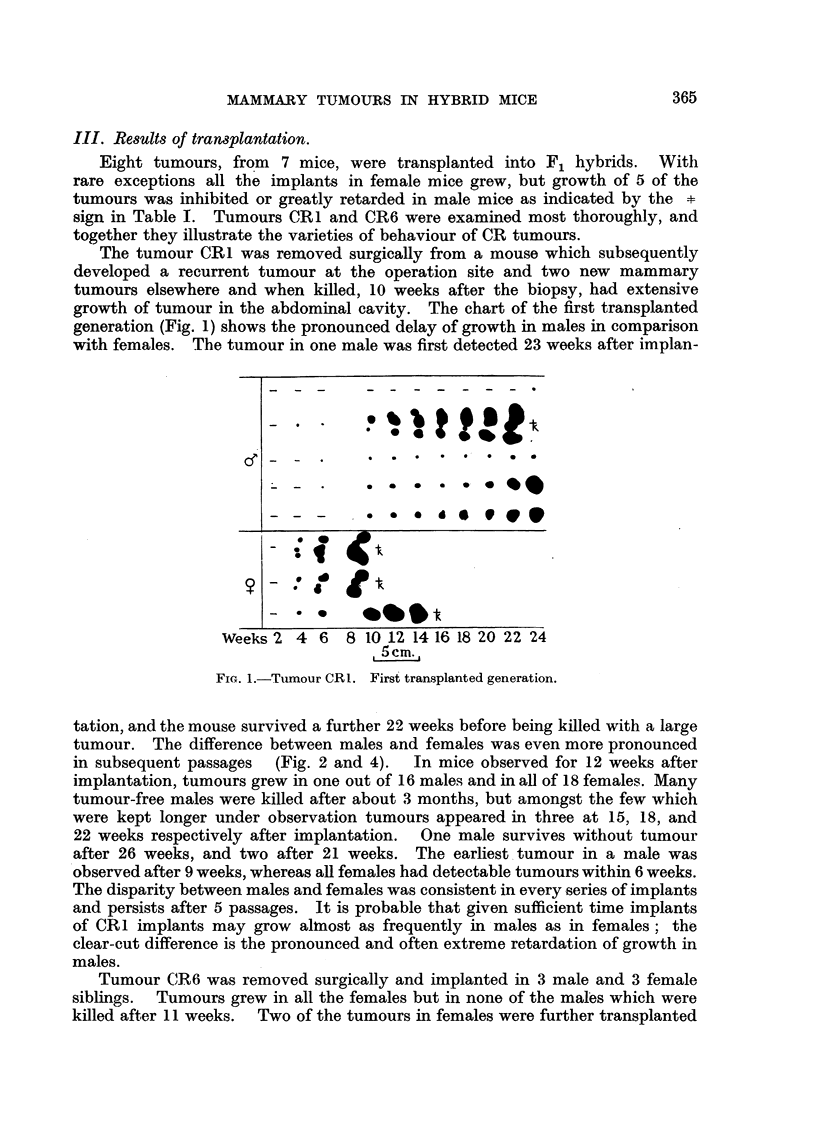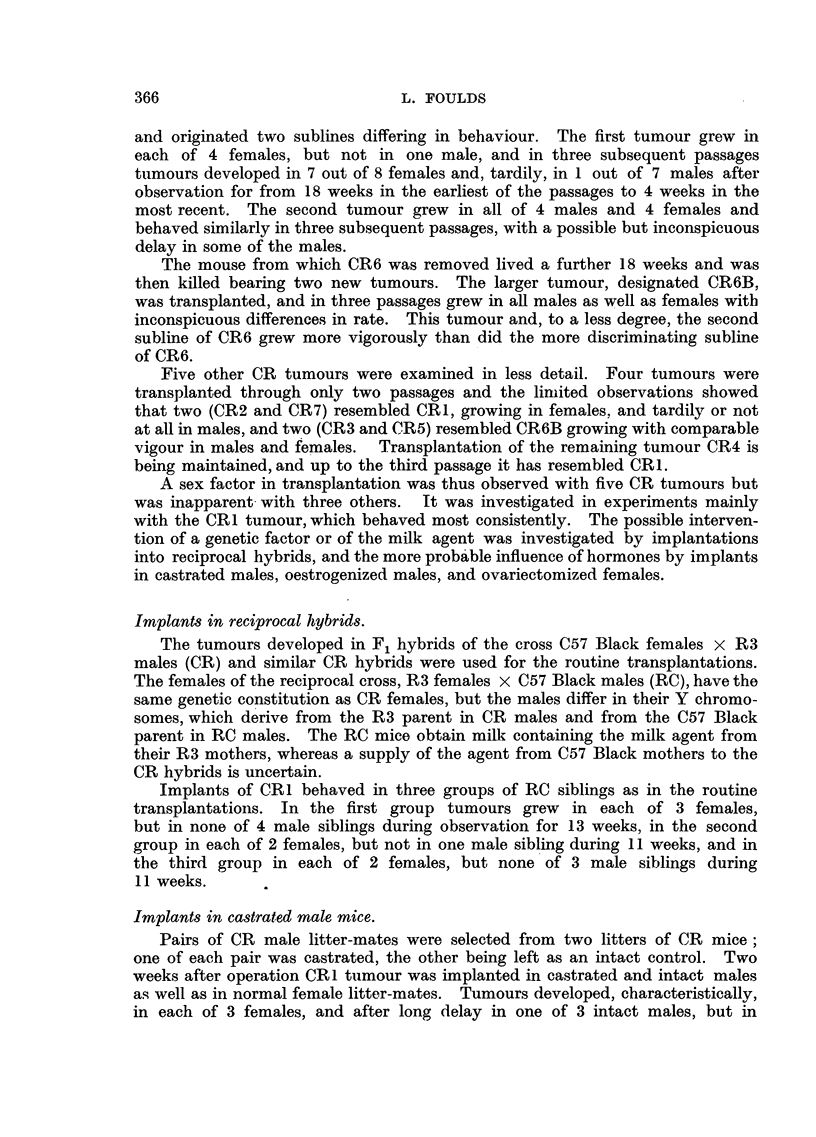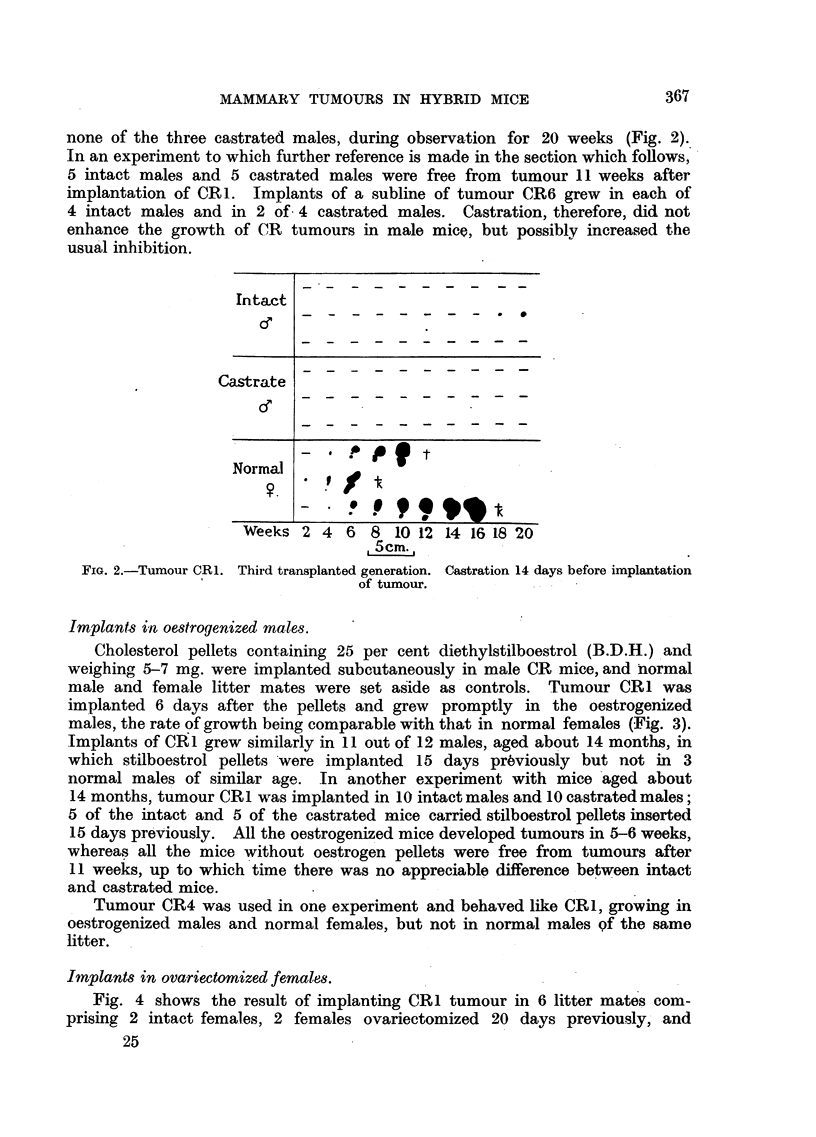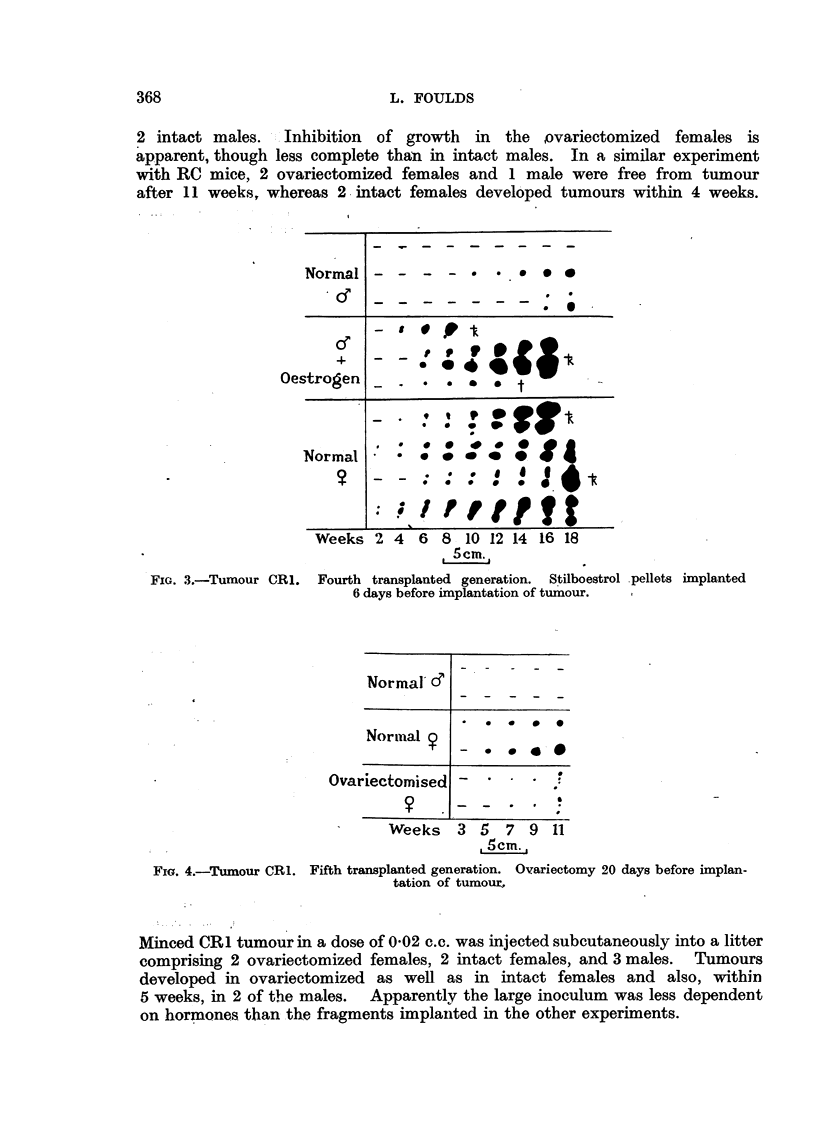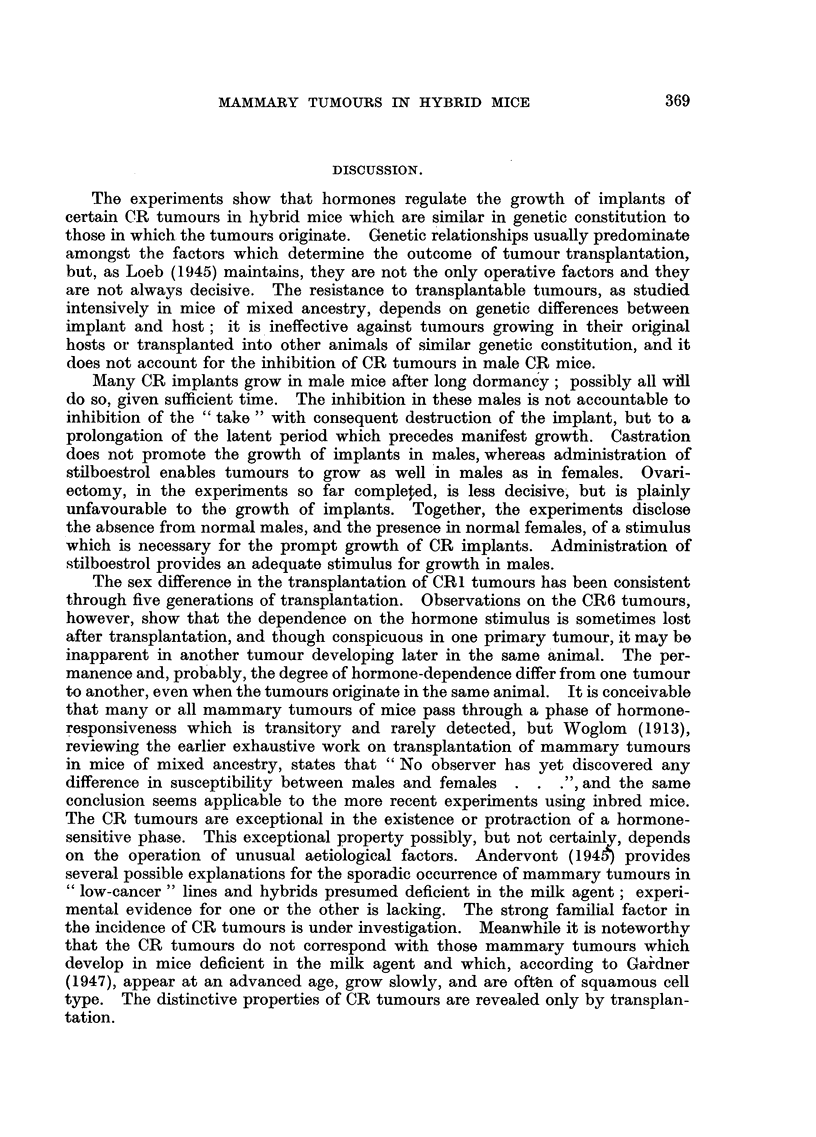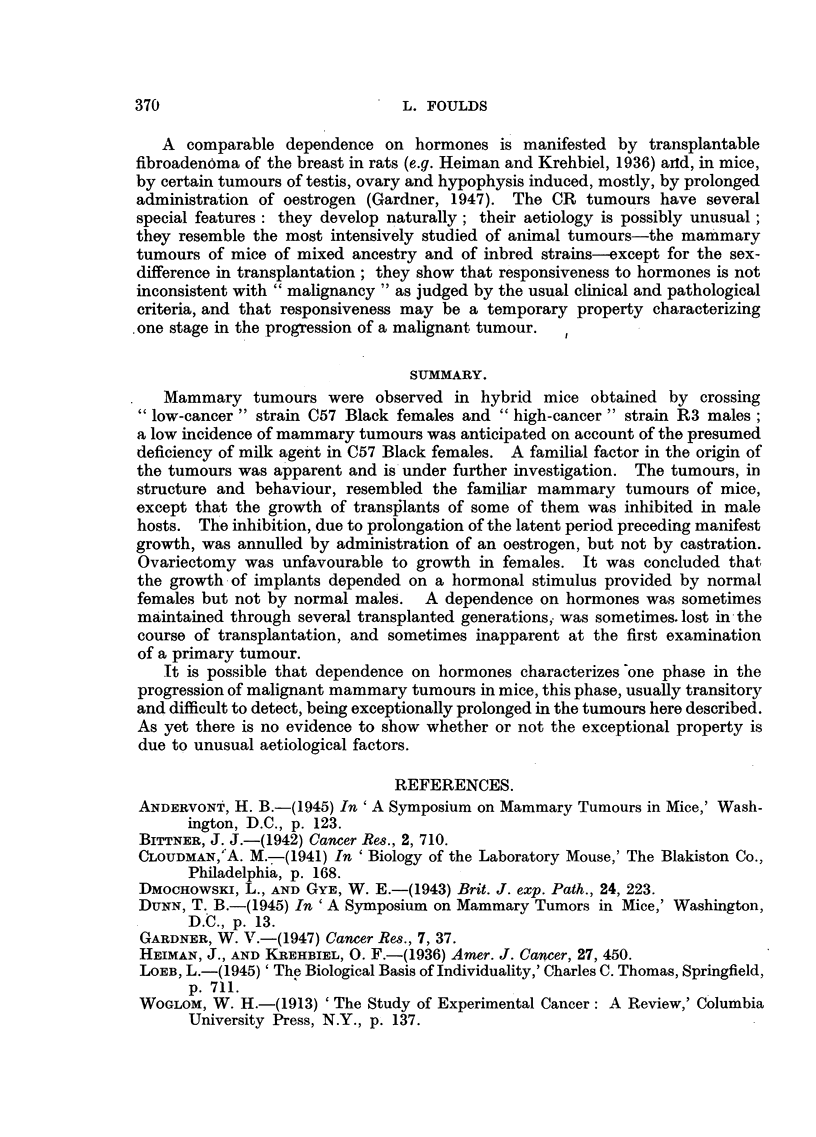# Mammary Tumours in Hybrid Mice: A Sex-factor in Transplantation

**DOI:** 10.1038/bjc.1947.33

**Published:** 1947-12

**Authors:** L. Foulds


### MAMMARY TUMOURS IN HYBRID MICE: A SEX-FACTOR IN TRANSPLANTATION.

L. FOULDS.

*From the Laboratories of the Imperial Cancer Research Fund, London, N.W. 7.*

Received for publication October 9, 1947.

For many experiments hybrid mice obtained by crossing two inbred strains are preferable to either of the contributory pure lines. The $F_1$ hybrids, although not homozygous, are uniform in genetic constitution, they manifest "hybrid vigour," and reciprocal crosses permit the study of different maternal factors in conjunction with the same genotype. Mammary tumours develop unexpectedly and erratically in some hybrid and pure line mice supposedly deficient in the maternal milk agent which, according to Bittner (1942) and others, co-operates with oestrogenic stimulation and appropriate genetic constitution to evoke mammary tumours in mice of "high cancer" strains. The mammary tumours described in this paper developed in hybrid mice which were presumed deficient in the milk agent. Several of the tumours were readily transplantable into female but not into male mice of appropriate genetic constitution, and experiments showed that hormones controlled the outcome of transplantation.

### MATERIAL AND METHODS.

A breeding colony consisting of twelve C57 Black females and three R3 males was established in September, 1945. The C57 Black strain has been maintained in this laboratory by brother-sister matings since 1941 ; spontaneous mammary tumours have not been recorded in the pedigreed stock. The R3 strain has been similarly maintained since 1934, and Dmochowski and Gye (1943) recorded mammary tumours in 83 per cent of breeding females at an average age of 8·5 months. The presumed deficiency of milk agent in the C57 mothers gave reason to expect a low incidence of mammary tumours in the $F_1$ hybrids of the cross C57 Black females × R3 males (designated, shortly, CR). Mammary tumours developed, however, and their incidence was studied in $F_1$ hybrids born between January and July, 1946. During this period, litters were recorded and the mice, with few exceptions, were sexed and given identification marks at the time of weaning. Some females were kept as virgins but most were subjected to forced breeding, being continuously with males and deprived of their litters within 48 hours of birth. Some of the mice were used in fruitless experiments, particularly for intraperitoneal injections of small doses of methylcholanthrene or for transplantation experiments. The remainder suffered no experimental interference.

Tumours were registered serially as CR1, CR2, etc. Many were removed surgically. Material so obtained as well as post-mortem material was transplanted into $F_1$ hybrids similar to those having the primary tumours, but mostly from a different colony. Fragments of tumour were implanted subcutaneously in the flank by means of a transplanting needle. In one experiment minced tissue was injected, using a Bashford transplanting syringe. The mice used in routine transplantations were usually siblings, but for some experiments two or more litters were combined to provide requisite numbers of each sex. The growth of tumours was recorded regularly on silhouette charts.

### RESULTS.

### *I. Occurrence of tumours.*

Nine out of 71 breeding females which survived more than 6 months developed mammary tumours, and 48 are alive without tumours. One virgin female out of 22 which survived a similar period developed a tumour, and 21 are alive without tumours. The ages of the surviving mice range from about 14 to 20 months. No new tumours have been observed during the past 6 months. Particulars of the tumours are summarized in Table I, where the tumour-bearing mice are arranged in order of birth.

The distribution of the tumours suggests a strong familial factor in their incidence. Each of the four female mice of Litter 46/3 developed a tumour, as did each of the two in Litter 46/28. Two litters, 46/33 and 46/34 were mixed before the mice were marked ; consequently the mice bearing tumours CR4 and CR10 were possibly, but not certainly, sisters. Tumour CR6 developed in the only female of Litter 46/35 which survived more than 6 months.

Table I shows the age of the mice when tumours were first observed. The average tumour age was 35·8 weeks for the whole group and 33·7 weeks for the breeding females, the latter figure being almost identical with that recorded by

TABLE I.

| Serial No. | Litter No. | Tumour-age (weeks). | Transplantation. ♀ | Transplantation. ♂ | |
|---|---|---|---|---|---|
| CR1 | 46/3 | 39 | + | ǂ | .. |
| CR2 | 46/3 | 39 | + | ǂ | .. |
| CR3 | 46/3 | 44 | + | + | One I.P. injection of methylcholanthrene. |
| CR5 | 46/3 | 46 | + | + | One I.P. injection of methylcholanthrene. |
| CR9 | 46/6 | 55 | .... | .... | Virgin. One I.P. injection of methylcholanthrene. |
| CR7 | 46/28 | 34 | + | ǂ | .. |
| CR8 | 46/28 | 28 | .... | .... | .. |
| CR4 | 46/33–34 | 15 | + | ǂ | .. |
| CR10 | 46/33–34 | 26 | .... | .... | .. |
| CR6 | 46/35 | 24 | + | ǂ | Biopsy. |
| CR6B | 46/35 | 39 | + | + | Post-mortem. |

Dmochowski and Gye for R3 breeding females. The appearance of CR4 at the early age of 15 weeks is noteworthy.

Tumours developed in three mice (CR3, CR5 and CR9) which had received each one intraperitoneal injection of 0·25 c.c. of a 0·5 per cent solution of methylcholanthrene in sesame oil. It is improbable that the carcinogen induced or accelerated tumours CR3 and CR5, for tumours CR1 and CR2 developed, without carcinogen, in sibling mice at a somewhat earlier age. The effect of the carcinogen on the growth of tumour CR9 in a virgin is conjectural. Tumour CR10 developed in a mouse bearing a slow-growing implant of tumour CR1. The remaining 6 tumour-bearing mice had not been subjected to experiment.

*II. Characteristics of the tumours.*

The tumours were not grossly or microscopically different from the familiar mammary tumours of "high-cancer" strains or of mice of mixed ancestry as described by Cloudman (1941) and Dunn (1945). Eight tumours were removed surgically from 4 mice and 6 recurred locally. Before death 7 of the 10 tumour-bearing mice had multiple tumours. Two tumours (CR4 and CR8) regressed under observation, but soon recurred. Post-mortem examination disclosed extensive intraperitoneal dissemination of CR1. Metastatic deposits were not found. The histological structure of the tumours was unremarkable; in particular, squamous differentiation was found, in a slight degree, in only one of the many sections examined.

Transplanted tumours were likewise unremarkable in growth and structure. Secondary tumours were found in the lungs of mice bearing tumours CR1, CR3 and CR6. Some tumours exuded milky fluid on section and microscopical examination revealed pronounced secretory activity; otherwise the microscopical appearances were not distinctive.

*III. Results of transplantation.*

Eight tumours, from 7 mice, were transplanted into $F_1$ hybrids. With rare exceptions all the implants in female mice grew, but growth of 5 of the tumours was inhibited or greatly retarded in male mice as indicated by the ≠ sign in Table I. Tumours CR1 and CR6 were examined most thoroughly, and together they illustrate the varieties of behaviour of CR tumours.

The tumour CR1 was removed surgically from a mouse which subsequently developed a recurrent tumour at the operation site and two new mammary tumours elsewhere and when killed, 10 weeks after the biopsy, had extensive growth of tumour in the abdominal cavity. The chart of the first transplanted generation (Fig. 1) shows the pronounced delay of growth in males in comparison with females. The tumour in one male was first detected 23 weeks after implan-

FIG. 1.—Tumour CR1. First transplanted generation.

tation, and the mouse survived a further 22 weeks before being killed with a large tumour. The difference between males and females was even more pronounced in subsequent passages (Fig. 2 and 4). In mice observed for 12 weeks after implantation, tumours grew in one out of 16 males and in all of 18 females. Many tumour-free males were killed after about 3 months, but amongst the few which were kept longer under observation tumours appeared in three at 15, 18, and 22 weeks respectively after implantation. One male survives without tumour after 26 weeks, and two after 21 weeks. The earliest tumour in a male was observed after 9 weeks, whereas all females had detectable tumours within 6 weeks. The disparity between males and females was consistent in every series of implants and persists after 5 passages. It is probable that given sufficient time implants of CR1 implants may grow almost as frequently in males as in females ; the clear-cut difference is the pronounced and often extreme retardation of growth in males.

Tumour CR6 was removed surgically and implanted in 3 male and 3 female siblings. Tumours grew in all the females but in none of the males which were killed after 11 weeks. Two of the tumours in females were further transplanted and originated two sublines differing in behaviour. The first tumour grew in each of 4 females, but not in one male, and in three subsequent passages tumours developed in 7 out of 8 females and, tardily, in 1 out of 7 males after observation for from 18 weeks in the earliest of the passages to 4 weeks in the most recent. The second tumour grew in all of 4 males and 4 females and behaved similarly in three subsequent passages, with a possible but inconspicuous delay in some of the males.

The mouse from which CR6 was removed lived a further 18 weeks and was then killed bearing two new tumours. The larger tumour, designated CR6B, was transplanted, and in three passages grew in all males as well as females with inconspicuous differences in rate. This tumour and, to a less degree, the second subline of CR6 grew more vigorously than did the more discriminating subline of CR6.

Five other CR tumours were examined in less detail. Four tumours were transplanted through only two passages and the limited observations showed that two (CR2 and CR7) resembled CR1, growing in females, and tardily or not at all in males, and two (CR3 and CR5) resembled CR6B growing with comparable vigour in males and females. Transplantation of the remaining tumour CR4 is being maintained, and up to the third passage it has resembled CR1.

A sex factor in transplantation was thus observed with five CR tumours but was inapparent with three others. It was investigated in experiments mainly with the CR1 tumour, which behaved most consistently. The possible intervention of a genetic factor or of the milk agent was investigated by implantations into reciprocal hybrids, and the more probable influence of hormones by implants in castrated males, oestrogenized males, and ovariectomized females.

*Implants in reciprocal hybrids.*

The tumours developed in $F_1$ hybrids of the cross C57 Black females $\times$ R3 males (CR) and similar CR hybrids were used for the routine transplantations. The females of the reciprocal cross, R3 females $\times$ C57 Black males (RC), have the same genetic constitution as CR females, but the males differ in their Y chromosomes, which derive from the R3 parent in CR males and from the C57 Black parent in RC males. The RC mice obtain milk containing the milk agent from their R3 mothers, whereas a supply of the agent from C57 Black mothers to the CR hybrids is uncertain.

Implants of CR1 behaved in three groups of RC siblings as in the routine transplantations. In the first group tumours grew in each of 3 females, but in none of 4 male siblings during observation for 13 weeks, in the second group in each of 2 females, but not in one male sibling during 11 weeks, and in the third group in each of 2 females, but none of 3 male siblings during 11 weeks.

*Implants in castrated male mice.*

Pairs of CR male litter-mates were selected from two litters of CR mice ; one of each pair was castrated, the other being left as an intact control. Two weeks after operation CR1 tumour was implanted in castrated and intact males as well as in normal female litter-mates. Tumours developed, characteristically, in each of 3 females, and after long delay in one of 3 intact males, but in none of the three castrated males, during observation for 20 weeks (Fig. 2). In an experiment to which further reference is made in the section which follows, 5 intact males and 5 castrated males were free from tumour 11 weeks after implantation of CR1. Implants of a subline of tumour CR6 grew in each of 4 intact males and in 2 of 4 castrated males. Castration, therefore, did not enhance the growth of CR tumours in male mice, but possibly increased the usual inhibition.

FIG. 2.—Tumour CR1. Third transplanted generation. Castration 14 days before implantation of tumour.

*Implants in oestrogenized males.*

Cholesterol pellets containing 25 per cent diethylstilboestrol (B.D.H.) and weighing 5–7 mg. were implanted subcutaneously in male CR mice, and normal male and female litter mates were set aside as controls. Tumour CR1 was implanted 6 days after the pellets and grew promptly in the oestrogenized males, the rate of growth being comparable with that in normal females (Fig. 3). Implants of CR1 grew similarly in 11 out of 12 males, aged about 14 months, in which stilboestrol pellets were implanted 15 days previously but not in 3 normal males of similar age. In another experiment with mice aged about 14 months, tumour CR1 was implanted in 10 intact males and 10 castrated males; 5 of the intact and 5 of the castrated mice carried stilboestrol pellets inserted 15 days previously. All the oestrogenized mice developed tumours in 5–6 weeks, whereas all the mice without oestrogen pellets were free from tumours after 11 weeks, up to which time there was no appreciable difference between intact and castrated mice.

Tumour CR4 was used in one experiment and behaved like CR1, growing in oestrogenized males and normal females, but not in normal males of the same litter.

*Implants in ovariectomized females.*

Fig. 4 shows the result of implanting CR1 tumour in 6 litter mates comprising 2 intact females, 2 females ovariectomized 20 days previously, and

2 intact males. Inhibition of growth in the ovariectomized females is apparent, though less complete than in intact males. In a similar experiment with RC mice, 2 ovariectomized females and 1 male were free from tumour after 11 weeks, whereas 2 intact females developed tumours within 4 weeks.

FIG. 3.—Tumour CR1. Fourth transplanted generation. Stilboestrol pellets implanted 6 days before implantation of tumour.

FIG. 4.—Tumour CR1. Fifth transplanted generation. Ovariectomy 20 days before implantation of tumour.

Minced CR1 tumour in a dose of 0·02 c.c. was injected subcutaneously into a litter comprising 2 ovariectomized females, 2 intact females, and 3 males. Tumours developed in ovariectomized as well as in intact females and also, within 5 weeks, in 2 of the males. Apparently the large inoculum was less dependent on hormones than the fragments implanted in the other experiments.

### DISCUSSION.

The experiments show that hormones regulate the growth of implants of certain CR tumours in hybrid mice which are similar in genetic constitution to those in which the tumours originate. Genetic relationships usually predominate amongst the factors which determine the outcome of tumour transplantation, but, as Loeb (1945) maintains, they are not the only operative factors and they are not always decisive. The resistance to transplantable tumours, as studied intensively in mice of mixed ancestry, depends on genetic differences between implant and host ; it is ineffective against tumours growing in their original hosts or transplanted into other animals of similar genetic constitution, and it does not account for the inhibition of CR tumours in male CR mice.

Many CR implants grow in male mice after long dormancy ; possibly all will do so, given sufficient time. The inhibition in these males is not accountable to inhibition of the " take " with consequent destruction of the implant, but to a prolongation of the latent period which precedes manifest growth. Castration does not promote the growth of implants in males, whereas administration of stilboestrol enables tumours to grow as well in males as in females. Ovariectomy, in the experiments so far completed, is less decisive, but is plainly unfavourable to the growth of implants. Together, the experiments disclose the absence from normal males, and the presence in normal females, of a stimulus which is necessary for the prompt growth of CR implants. Administration of stilboestrol provides an adequate stimulus for growth in males.

The sex difference in the transplantation of CR1 tumours has been consistent through five generations of transplantation. Observations on the CR6 tumours, however, show that the dependence on the hormone stimulus is sometimes lost after transplantation, and though conspicuous in one primary tumour, it may be inapparent in another tumour developing later in the same animal. The permanence and, probably, the degree of hormone-dependence differ from one tumour to another, even when the tumours originate in the same animal. It is conceivable that many or all mammary tumours of mice pass through a phase of hormone-responsiveness which is transitory and rarely detected, but Woglom (1913), reviewing the earlier exhaustive work on transplantation of mammary tumours in mice of mixed ancestry, states that " No observer has yet discovered any difference in susceptibility between males and females . . .", and the same conclusion seems applicable to the more recent experiments using inbred mice. The CR tumours are exceptional in the existence or protraction of a hormone-sensitive phase. This exceptional property possibly, but not certainly, depends on the operation of unusual aetiological factors. Andervont (1945) provides several possible explanations for the sporadic occurrence of mammary tumours in " low-cancer " lines and hybrids presumed deficient in the milk agent ; experimental evidence for one or the other is lacking. The strong familial factor in the incidence of CR tumours is under investigation. Meanwhile it is noteworthy that the CR tumours do not correspond with those mammary tumours which develop in mice deficient in the milk agent and which, according to Gardner (1947), appear at an advanced age, grow slowly, and are often of squamous cell type. The distinctive properties of CR tumours are revealed only by transplantation.

A comparable dependence on hormones is manifested by transplantable fibroadenoma of the breast in rats (*e.g.* Heiman and Krehbiel, 1936) and, in mice, by certain tumours of testis, ovary and hypophysis induced, mostly, by prolonged administration of oestrogen (Gardner, 1947). The CR tumours have several special features : they develop naturally ; their aetiology is possibly unusual ; they resemble the most intensively studied of animal tumours—the mammary tumours of mice of mixed ancestry and of inbred strains—except for the sex-difference in transplantation ; they show that responsiveness to hormones is not inconsistent with " malignancy " as judged by the usual clinical and pathological criteria, and that responsiveness may be a temporary property characterizing one stage in the progression of a malignant tumour.

SUMMARY.

Mammary tumours were observed in hybrid mice obtained by crossing " low-cancer " strain C57 Black females and " high-cancer " strain R3 males ; a low incidence of mammary tumours was anticipated on account of the presumed deficiency of milk agent in C57 Black females. A familial factor in the origin of the tumours was apparent and is under further investigation. The tumours, in structure and behaviour, resembled the familiar mammary tumours of mice, except that the growth of transplants of some of them was inhibited in male hosts. The inhibition, due to prolongation of the latent period preceding manifest growth, was annulled by administration of an oestrogen, but not by castration. Ovariectomy was unfavourable to growth in females. It was concluded that the growth of implants depended on a hormonal stimulus provided by normal females but not by normal males. A dependence on hormones was sometimes maintained through several transplanted generations, was sometimes lost in the course of transplantation, and sometimes inapparent at the first examination of a primary tumour.

It is possible that dependence on hormones characterizes one phase in the progression of malignant mammary tumours in mice, this phase, usually transitory and difficult to detect, being exceptionally prolonged in the tumours here described. As yet there is no evidence to show whether or not the exceptional property is due to unusual aetiological factors.

### REFERENCES.

ANDERVONT, H. B.—(1945) *In* ' A Symposium on Mammary Tumours in Mice,' Washington, D.C., p. 123.

BITTNER, J. J.—(1942) *Cancer Res.*, **2**, 710.

CLOUDMAN, A. M.—(1941) *In* ' Biology of the Laboratory Mouse,' The Blakiston Co., Philadelphia, p. 168.

DMOCHOWSKI, L., AND GYE, W. E.—(1943) *Brit. J. exp. Path.*, **24**, 223.

DUNN, T. B.—(1945) *In* ' A Symposium on Mammary Tumors in Mice,' Washington, D.C., p. 13.

GARDNER, W. V.—(1947) *Cancer Res.*, **7**, 37.

HEIMAN, J., AND KREHBIEL, O. F.—(1936) *Amer. J. Cancer*, **27**, 450.

LOEB, L.—(1945) ' The Biological Basis of Individuality,' Charles C. Thomas, Springfield, p. 711.

WOGLOM, W. H.—(1913) ' The Study of Experimental Cancer : A Review,' Columbia University Press, N.Y., p. 137.